# Short-duration atrial fibrillation in ischemic stroke: high risk despite subclinical burden-a prospective cohort study

**DOI:** 10.1186/s12872-025-05080-1

**Published:** 2025-08-20

**Authors:** Priyanka Boettger, Karolis Macius, Jamschid Sedighi, Henning Lemm, Kerstin Piayda, Bernhard Unsoeld, Samuel Sossalla, Omar Alhaj Omar, Martin Juenemann, Michael Buerke

**Affiliations:** 1 Department of Internal Medicine I, Cardiology, Angiology and Intensive Care, Justus-Liebig University Hospital, Klinikstrasse 33, Giessen, Germany; 2 Department of Neurology, Justus-Liebig University, Giessen, Germany; 3 Department of Internal Medicine II, Cardiology, Angiology and Intensive Care Medicine, Heart-Center-Südwestfalen, Siegen, Germany

**Keywords:** Atrial fibrillation, Electrocardiography, Stroke/etiology, Risk assessment, Ischemic stroke, Prospective studies

## Abstract

**Background and purpose:**

Atrial fibrillation (AF) episodes ≥ 30 s are currently considered clinically relevant in stroke diagnostics. However, shorter AF episodes may signal a significant embolic risk, especially in patients with embolic stroke of undetermined source (ESUS). This study investigates the prevalence, risk profile, and stroke severity associated with short-duration AF (SDAF < 30 s) across ischemic stroke subtypes.

**Methods:**

We prospectively enrolled 714 consecutive patients with ischemic stroke or Transient ischemic attacks who underwent ≥ 48-hour ECG monitoring. AF episodes were classified as 0–14 s, 15–29 s, or ≥ 30 s. Stroke subtypes were defined using TOAST and ESUS criteria. Risk profiles, NIH Stroke Scale scores, and CHA₂DS₂-VASc scores were analyzed by AF duration.

**Results:**

AF of any duration was detected in 53.8% of patients; 22.8% had episodes ≥ 30 s and 29.9% had SDAF. Among ESUS patients, 35.7% exhibited SDAF, and 80.2% of these had CHA₂DS₂-VASc scores ≥ 2. Stroke severity and risk scores were significantly higher in patients with SDAF than those without AF. SDAF was more prevalent in women (37.0%) and in individuals aged > 65 years (89.4%).

**Conclusions:**

SDAF is common across stroke subtypes—particularly ESUS—and is associated with elevated thromboembolic risk despite falling below current diagnostic thresholds. These findings highlight a diagnostic blind spot in stroke workup and support reevaluation of duration-based criteria for post-stroke AF detection and risk profiling.

**Supplementary Information:**

The online version contains supplementary material available at 10.1186/s12872-025-05080-1.

## Background and purpose

Current stroke guidelines consider atrial fibrillation (AF) clinically actionable only when episodes last ≥ 30 s, a definition supported by device-based studies and consensus guidelines [1–4]. However, shorter episodes are increasingly observed during post-stroke monitoring and may indicate a clinically meaningful arrhythmic burden [5–7]. In particular, cryptogenic stroke and ESUS frequently lack an identified embolic source despite extended diagnostic workup [8, 9]. Real-world data suggest that short-duration AF (SDAF) may correlate with age, stroke severity, and CHA₂DS₂-VASc scores [10]raising concern that these brief episodes, though guideline-excluded, could represent a high-risk but undertreated subgroup. In this prospective cohort study, we aimed to assess the prevalence of SDAF (< 30 s) across ischemic stroke subtypes, determine associated risk profiles, and evaluate stroke severity. We hypothesized that SDAF is common in ESUS and cryptogenic stroke and is associated with elevated thromboembolic risk despite not fulfilling current diagnostic thresholds—thereby representing a diagnostic blind spot in contemporary stroke care.

## Methods

Over six months, all consecutive patients presenting with suspected ischemic stroke or Transient ischemic attack (TIA) were prospectively enrolled. Adults aged ≥ 18 years with acute ischemic stroke or TIA were eligible. Patients with hemorrhagic stroke or hospital stays < 24 h were excluded. Demographics, comorbidities, and diagnostic data were systematically recorded.

Stroke subtypes were classified using TOAST [11] and ESUS [8] criteria, aligned with current prevention and treatment guidelines [12]. Subtypes included: (1) large-artery atherosclerosis, defined by ≥ 50% stenosis (NASCET) or occlusion of relevant vessels; (2) cardioembolism, including AF, atrial flutter, intracardiac thrombus, valvular heart disease, recent myocardial infraction, LVEF < 35%, or endocarditis; (3) small vessel disease, defined by deep lacunar infarcts ≤ 15 mm (CT) or ≤ 20 mm (MRI); (4) other determined causes, including vasculitis, dissection, hematologic, storage, or mitochondrial disorders; and (5) ESUS, when no cause was identified after standard workup.

TIA was defined as a transient neurological deficit lasting < 24 h without imaging evidence of infarction, in accordance with the tissue-based definition proposed by Albers et al. [13, 14] Stroke workup included CT or MRI, extracranial/intracranial Doppler, transthoracic or transesophageal echocardiography, ≥ 48-hour ECG monitoring, and labs. Neurological status was assessed using the NIH Stroke Scale (NIHSS) at admission and every six hours. All patients received a 12-lead ECG at admission, followed by ≥ 48-hour Holter ECG or continuous telemetry [15]. No implantable loop recorders were used. AF episodes were categorized as < 30 s (0–14 s, 15–29 s) or ≥ 30 s. AF episodes lasting ≥ 30 s were considered diagnostic of AF, based on current guideline recommendations. [2, 16] Cardiovascular risk factors and CHA₂DS₂-VASc scores were recorded.

## Statistics

Although no formal a priori power calculation was performed,exploratory hypothesis testing and multivariable regression analyses were conducted toassess associations between SDAF and clinical characteristics. Categorical variables are presented as counts and percentages; continuous variables as means ± SD or medians with interquartile range (IQR, 25th–75th percentile), depending on distribution. Group comparisons were exploratory and used chi-square or Fisher’s exact test for categorical data, and t-test or Mann–Whitney U test for continuous variables. Odds ratios (OR) with 95% confidence intervals (CI) and p-values were calculated for key subgroup associations using SPSS^®^ (IBM). Post-hoc power analysis was performed using observed proportions, sample sizes, and chi-square tests via the statsmodels package in Python. Descriptive graphics were generated using Microsoft Excel^®^. In addition, an exploratory multivariable logistic regression analysis was performed to identify independent predictors of short-duration atrial fibrillation, including age, sex, stroke subtype, and hypertension.

## Results

During the six-month period, 771 patients with suspected stroke were admitted. After excluding 57 with hemorrhagic stroke, 714 patients were included in the final analysis. The mean age of the cohort was 73.2 ± 9.1 years, and the mean NIHSS on admission was 4.8 ± 3.6. Of these, 185 (25.9%) had TIA, 209 (29.3%) cardioembolic stroke, 110 (20.8%) large-artery atherosclerotic stroke, 40 (7.6%) lacunar stroke, and 163 (22.8%) cryptogenic stroke according to TOAST, with 98 (13.7%) meeting criteria for ESUS. The prevalence of hypertension was significantly higher among patients with short-duration AF compared to those without (82% vs. 71%, *p* = 0.002), a difference that remained significant after adjustment for age and sex (adjusted OR 1.75; 95% CI, 1.20–2.55; *p* = 0.004). Further baseline characteristics can be found in Table 1.

**Table 1:** Baseline characteristics of patients with ischemic stroke, stratified by stroke subtype

	Stroke population	TIA	Cryptogenic	ESUS	Athero-sclerotic	Cardioembolic	Lacunar
	*n* = 714	*n* = 185 (25.9%)	*n* = 163 (30.8%)	*n* = 98 (18.5%)	*n* = 110 (20.8%)	*n* = 209 (29.3%)	*n* = 40 (7.6%)
Age, mean ± SD (range)	71 ± 9.2 (20–99)	72 ± 8.5 (20–95)	67 ± 10.1 (29–99)	67 ± 9.8 (29–81)	70 ± 8.7 (39–96)	75 ± 7.9 (31–98)	72 ± 8.3 (29–92)
Age, median (IQR)	74 (67–81)	75 (68–82)	69 (62–76)	70 (64–77)	69 (61–75)	77 (71–83)	77 (70–84)
NIHSS at admission, median (IQR)	7 (3–11)	2 (1–4)	7 (4–10)	6 (3–9)	8 (5–12)	11 (6–16)	4 (2–6)
NIHSS at discharge, median (IQR)	3 (1–5)	0 (0–1)	3 (1–4)	2 (1–3)	4 (2–6)	5 (3–7)	1 (0–2)
Comorbidities							
Previous TIA/stroke	181 (25.4%)	41 (22.2%)	37 (22.7%)	11 (11,2%)	36 (32.7%)	55 (26.3%)	10 (25.0%)
BMI, mean ± SD	29.1 ± 4.2	28.7 ± 4.5	30.2 ± 3.9	30.4 ± 4.1	28.9 ± 3.8	29.5 ± 4.4	29.3 ± 3.6
Obesity (BMI > 30 kg/m2)	334 (46,8%)	75 (40.5%)	84 (51.5%)	53 (54,2%)	45 (41.0%)	106 (50.7%)	19 (47,5%)
Hypertension	537 (75.2%)	151 (81.6%)	116 (71%)	71 (72,2%)	83 (72.4%)	156 (74.6%)	29(72.5%)
Diabetes mellitus	212	43 (23.2%)	47 (28.8%)	31 (31,6%)	39 (35.5%)	66 (31.6%)	16 (40.0%)
Atrial fibrillation	163 (22.8%)	42 (22.7%)	0 (0.0%)	0 (0%)	14 (12.7%)	101 (48.3%)	5 (12.5%)
Coronary artery disease	191 (26.8%)	41 (22.2%)	46 (28.2%)	27 (27,5%)	30 (27.3%)	64 (30.6%)	9 (22.5%)
Artificial heart valve	41 (5.7%)	6 (3.2%)	0 (0.0%)	0 (0%)	4 (3.6%)	30 (14.3%)	1 (2.5%)
Heart failure	66 (9%)	6 (2.7%)	14 (7.3%)	6 (6,1%)	5 (4.5%)	40 (19.3%)	1 (2.5%)
Hyper-cholesterinemia	274 (38.4%)	68 (36.8%)	66 (40.5%)	42 (42,9%)	59 (53.64%)	66 (31.6%)	16 (40.0%)
ECG monitoring							
Stroke unit monitoring, mean ± SD (h)	37.1 ± 6.8	30.6 ± 5.4	37.9 ± 7.1	36.0 ± 6.2	39.9 ± 7.5	42.5 ± 6.9	34.1 ± 5.7
Stroke Unit Monitoring	651 (91.2%)	165 (89.2%)	151 (92.6%)	94 (96,0%)	101 (91.8%)	191 (91.4%)	36 (90%)

Continuous variables are presented as mean ± SD or median with interquartile range (IQR); categorical variables as absolute numbers and percentages

*ESUS* Embolic stroke of undetermined source, *TIA* Transient ischemic attack, *NIHSS* National institutes of health stroke scale, *BMI* Body mass index, *ECG* Electrocardiogram

### Distribution of atrial fibrillation by stroke subtype

**Fig. 1:**
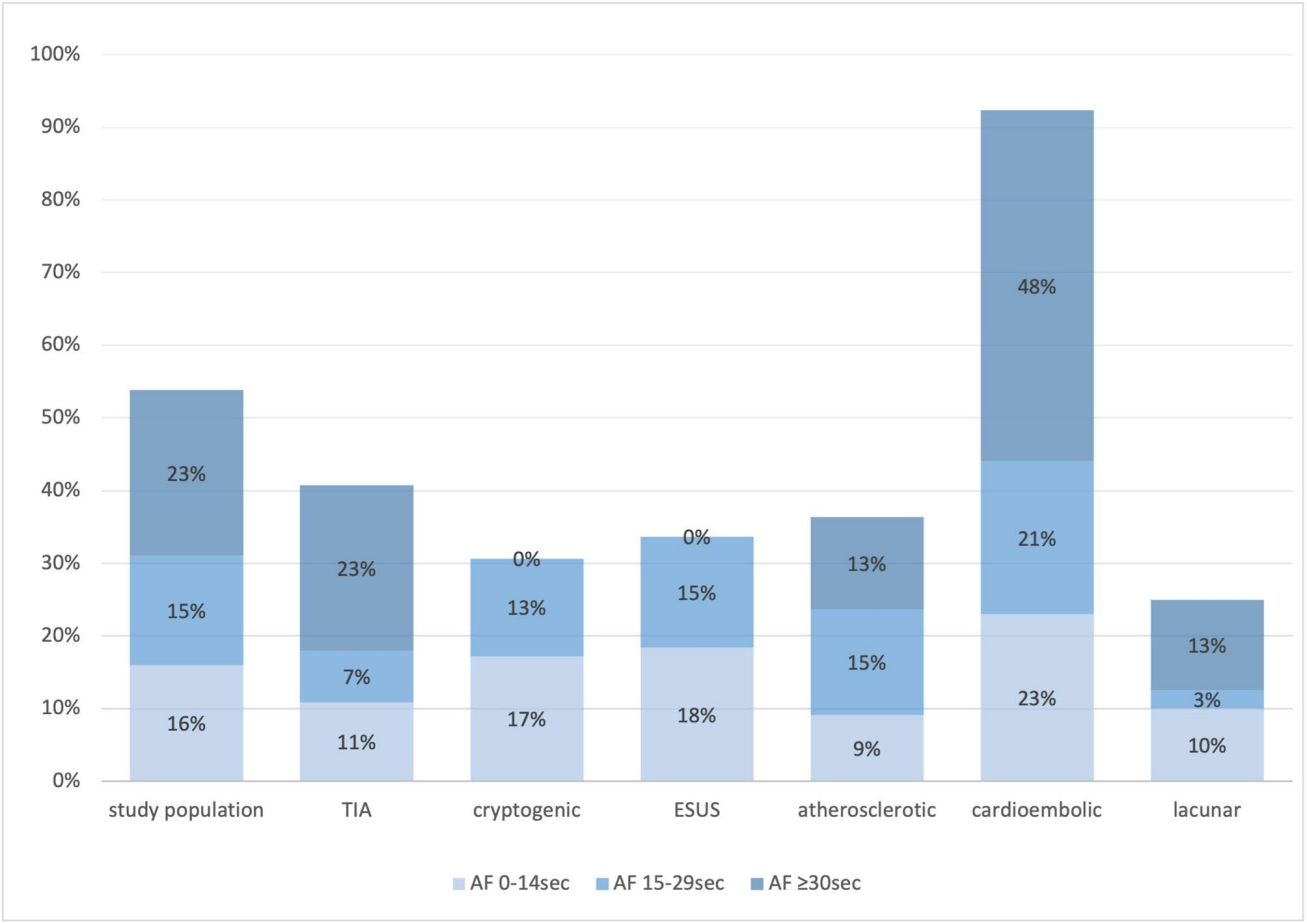
Distribution of atrial fibrillation episodes by ischemic stroke subtype. Prevalence of atrial fibrillation (AF) episodes ≥30 seconds, 15–29 seconds, and 0–14 seconds across stroke subtypes. AF of any duration was detected in 87.1% of cardioembolic strokes (n = 182/209), compared with 35.6% in cryptogenic stroke and 35.7% in ESUS. AF was least common in lacunar strokes (25.0%), with statistically significant differences across subtypes (*p*< 0.001). No patient with cryptogenic or ESUS stroke had AF ≥30 seconds

Atrial fibrillation (AF) of any duration was identified in 384 of 714 patients (53.8%). AF > 30 s occurred in 163 patients (22.8%), while 107 (15.0%) had AF episodes of 15–29 s and 114 (16.0%) had episodes of 0–14 s. AF distribution varied markedly across stroke subtypes (Fig. 1). In the cardioembolic stroke group (*n* = 209; mean age 78.2 ± 7.6 years), 94 patients (45.0%) exhibited AF > 30 s, 42 (20.1%) had episodes of 15–29 s, and 46 (22.0%) had 0–14 s episodes. Thus, 182 patients (87.1%) had AF of any duration—significantly more than in all other subtypes (OR 9.2; 95% CI, 6.2–13.6; *p* < 0.001). While the overall distribution of SDAF differed significantly across stroke subtypes (*p* < 0.001), the higher prevalence in ESUS compared to large-artery stroke did not reach statistical significance in direct comparison (*p* = 0.08), suggesting a trend that warrants further investigation. In cryptogenic stroke (*n* = 163; mean age 70.4 ± 8.1 years), no patient had AF > 30 s. However, 28 (17.2%) had AF episodes of 15–29 s and 30 (18.4%) of 0–14 s, yielding a short-duration AF prevalence of 35.6%. Similarly, among ESUS patients (*n* = 98), 18 (18.4%) had 15–29 s episodes and 17 (17.3%) had 0–14 s episodes, for a combined prevalence of 35.7%. No significant difference was found between cryptogenic and ESUS groups (*p* = 0.98). In TIA patients (*n* = 185; mean age 73.5 ± 9.4 years), AF > 30 s was seen in 41 (22.2%), while 13 (7.0%) and 18 (9.7%) had AF episodes of 15–29 and 0–14 s, respectively. Compared with the cardioembolic group, short-duration AF was significantly less common (OR 0.37; 95% CI, 0.23–0.60; *p* < 0.001). In atherosclerotic stroke (*n* = 110), AF > 30 s occurred in 13 patients (11.8%), with 17 (15.4%) and 10 (9.1%) having AF episodes of 15–29 and 0–14 s, respectively. Although short-duration AF was less frequent than in ESUS (25.4% vs. 35.7%), this difference was not statistically significant (*p* = 0.09). The lacunar stroke group (*n* = 40) had the lowest AF burden overall: 5 (12.5%) with AF > 30 s, 3 (7.5%) with 15–29 s episodes, and 2 (5.0%) with 0–14 s episodes. Compared to all other stroke subtypes, this difference reached statistical significance (*p* < 0.001).

### Age-related distribution of short-duration AF

**Fig. 2:**
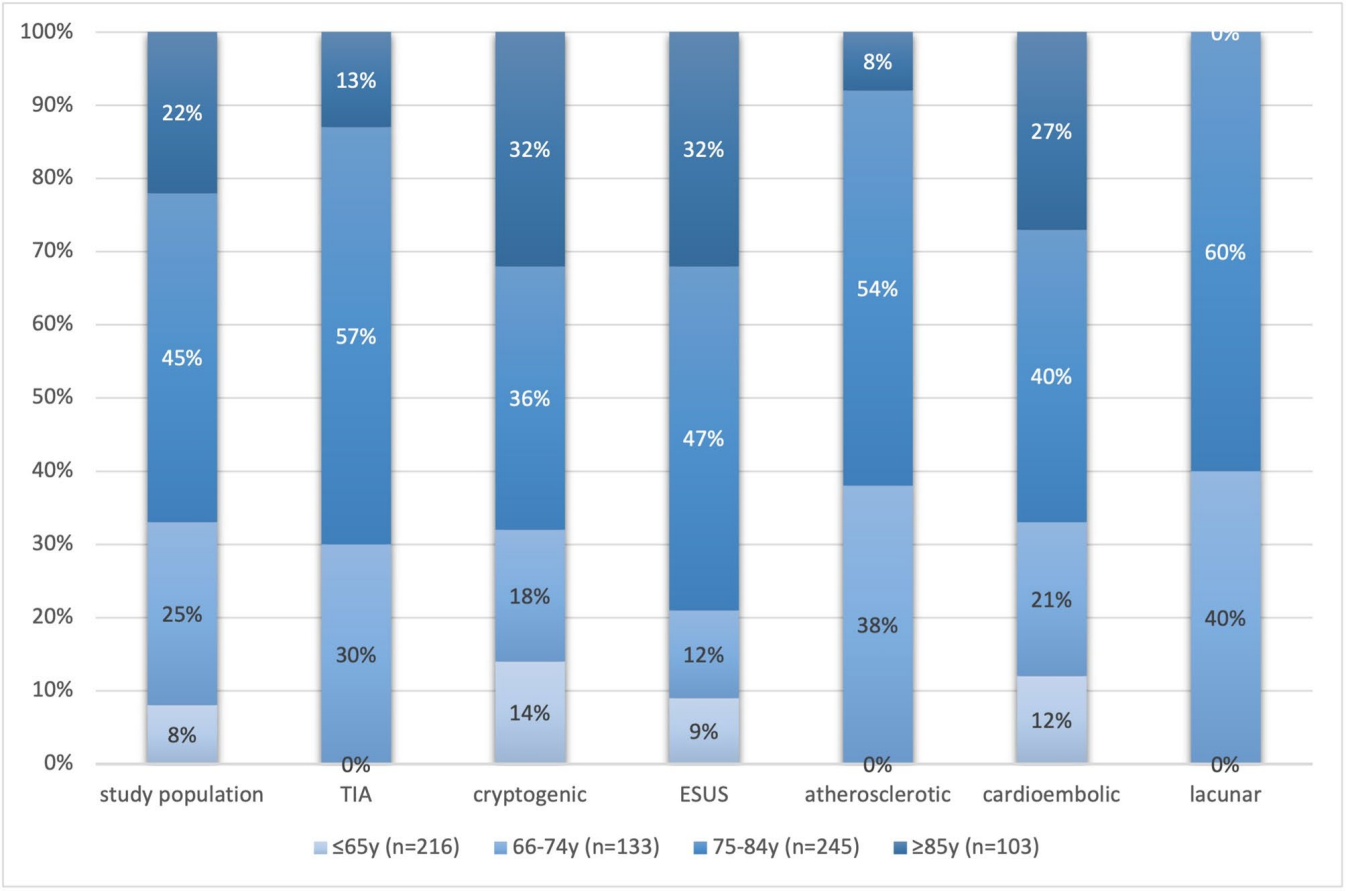
Age distribution of patients with short-duration atrial fibrillation. Short-duration AF was significantly more frequent in older patients: 36.8% in those aged 65–74 (49/133), 54.3% in those aged 75–84 (95/175), and 69.0% in those aged ≥85 years (40/58), with a significant trend across age strata (χ² for trend,*p*< 0.001). Overall, 89.4% of patients with SDAF were aged >65 years (OR 6.7; 95% CI, 4.3–10.5;*p*< 0.001)

Age was significantly associated with the presence of short-duration AF. Among the 207 patients with short-duration AF, 185 (89.4%) were aged > 65 years (mean age 78.1 ± 6.9 years), compared to 282 of 507 (55.6%) without short-duration AF (mean age 70.3 ± 8.4 years), yielding an odds ratio of 6.7 (95% CI, 4.3–10.5; *p* < 0.001). This age-related gradient was further supported by subgroup analysis: short-duration AF was present in 36.8% of individuals aged 65–74 (49/133), 54.3% in those aged 75–84 (95/175), and 69.0% in those ≥ 85 years (40/58) (Fig. 2). The trend across age strata was statistically significant (χ² for trend, *p* < 0.001).

### Sex differences in short-duration AF

**Fig. 3:**
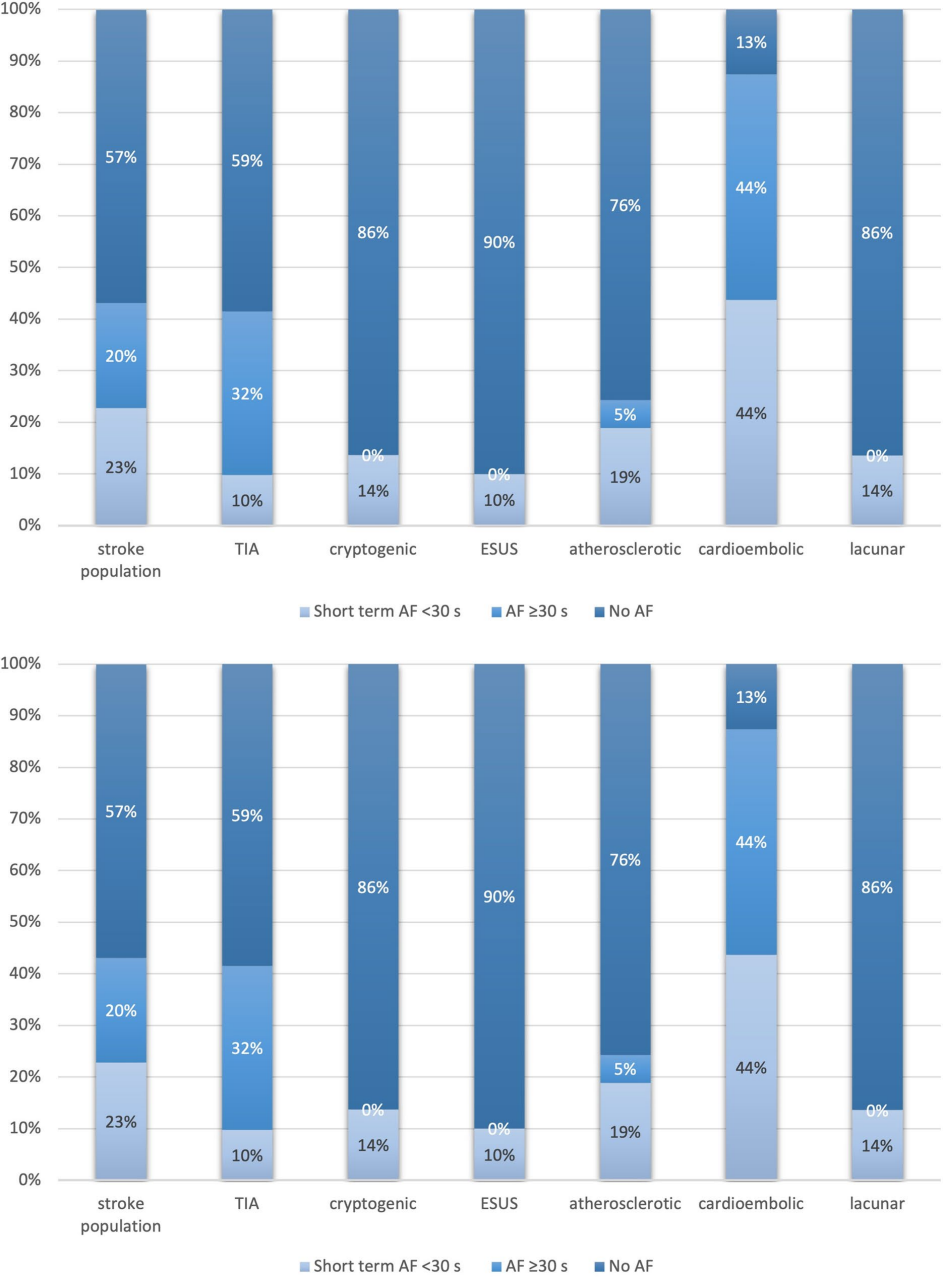
Sex-specific prevalence of short-duration atrial fibrillation after ischemic stroke. Panel (a) shows that 115 of 311 women (37.0%) exhibited SDAF, compared to 92 of 403 men (22.8%) in panel (b), yielding an odds ratio of 2.0 (95% CI, 1.4–2.9;*p*< 0.001). Women with SDAF also had significantly higher CHA₂DS₂-VASc scores than men (median 5 [IQR 4–6] vs. 4 [IQR 3–5];*p*< 0.001)

When assessing short-duration AF by sex, it was found to be more prevalent among female patients. Specifically, 115 out of 311 women (37.0%) experienced short-duration AF compared to 92 out of 403 men (22.8%). This difference was statistically significant, with an odds ratio of 2.0 (95% CI: 1.4–2.9, *p* < 0.001), indicating that women were nearly twice as likely to exhibit short-duration arrhythmias following stroke (Fig. 3a and b).

### Stroke severity and short-duration AF (NIHSS)

**Fig. 4:**
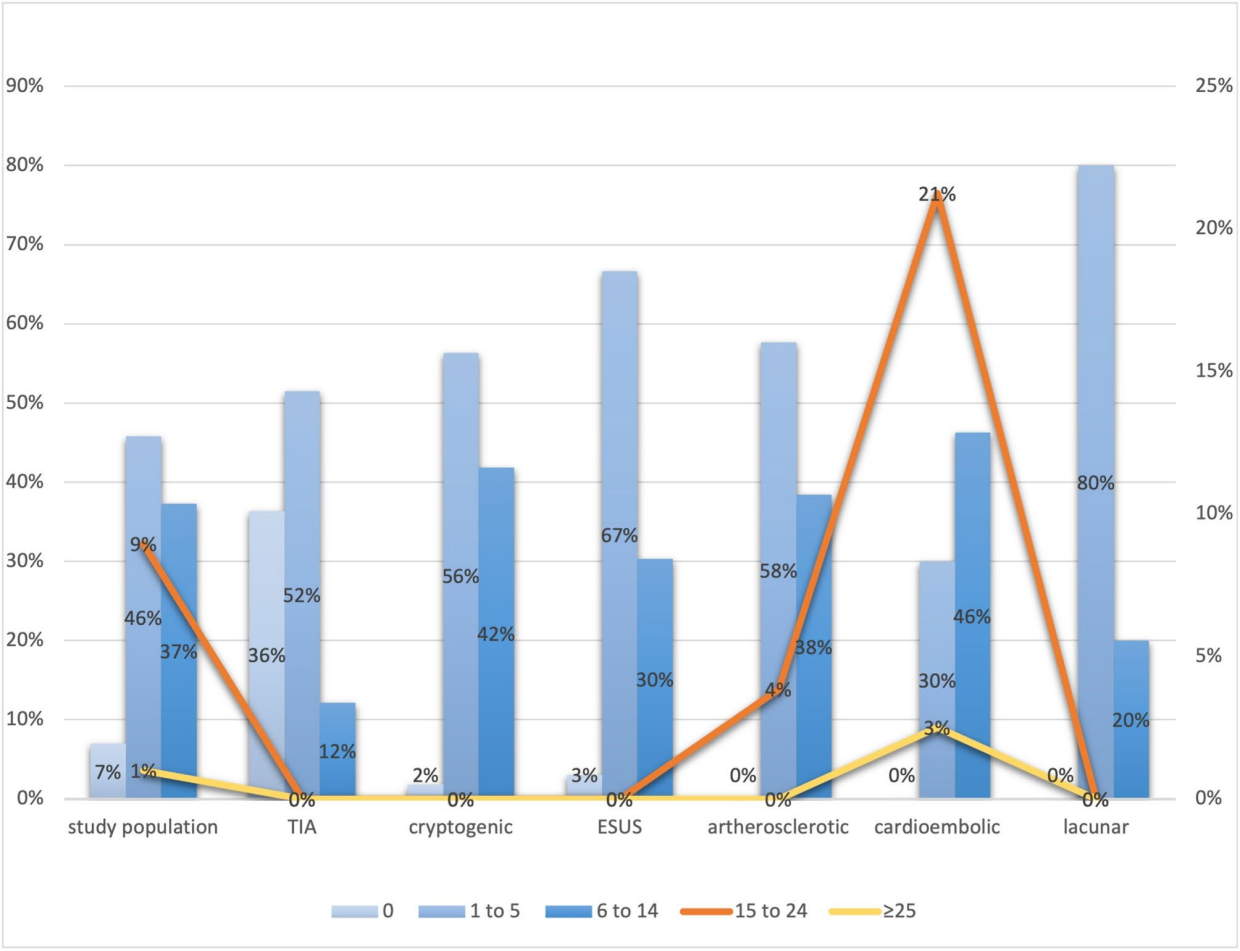
Stroke severity by atrial fibrillation status and duration. Median NIHSS scores on admission were significantly higher in patients with AF (median 6 [IQR 3–11]) compared to those without AF (3 [IQR 1–6];*p*< 0.001). Stroke severity increased with AF duration: median NIHSS was 7 [IQR 4–12] in AF ≥30 s and 4 [IQR 2–8] in SDAF (<30 s) (*p*= 0.003). A modest but significant correlation was observed between NIHSS and CHA₂DS₂-VASc scores in patients with SDAF (Spearman ρ = 0.23;*p*= 0.001)

Patients with any AF had more severe strokes than those without (median NIHSS 6 [IQR 3–11] vs. 3 [IQR 1–6]; *p* < 0.001). Severity increased with AF duration: NIHSS was higher in long-duration AF (≥ 30 s) than short-duration AF (median 7 [IQR 4–12] vs. 4 [IQR 2–8]; *p* = 0.003). In short-duration AF, NIHSS and CHA₂DS₂-VASc scores were modestly correlated (Spearman’s ρ = 0.23; *p* = 0.001). Stroke severity in short-duration AF varied by subtype (Fig. 4). TIA patients (*n* = 18, 9.7%) were typically neurologically intact (NIHSS 0), while most cryptogenic/ESUS patients (*n* = 58, 35.6%) had mild deficits (NIHSS 1–5). Cardioembolic stroke patients (*n* = 88, 42.1%) showed broader severity; 65.2% of all short-duration AF cases (*n* = 135) had NIHSS scores between 1 and 14. Mean NIHSS was higher in cardioembolic vs. ESUS patients with short-duration AF (8.3 ± 4.2 vs. 4.2 ± 2.8; *p* < 0.01).

### Premorbid CHA₂DS₂-VASc scores in short-duration AF and AF patients

**Fig. 5:**
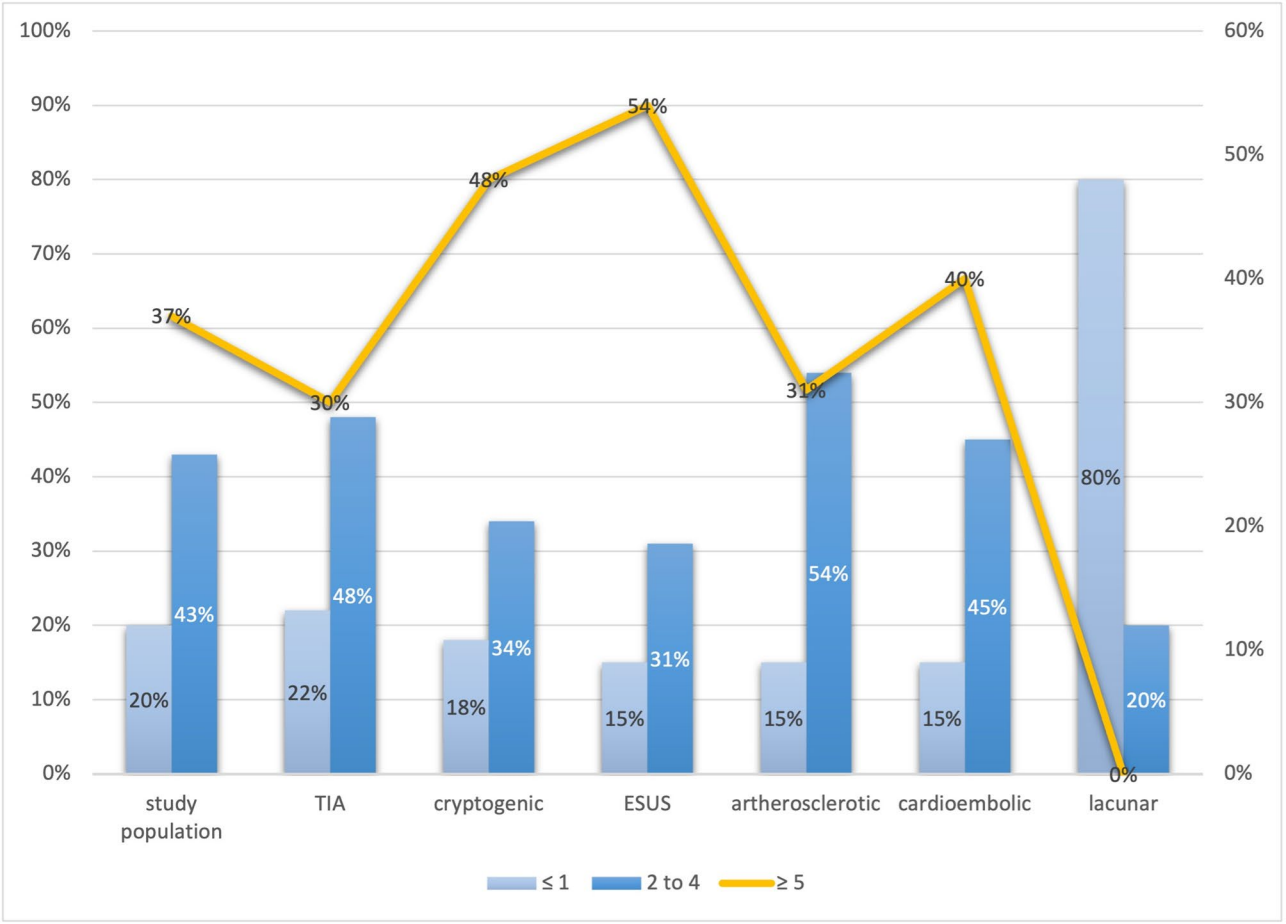
CHA₂DS₂-VASc score distribution in patients with short-duration atrial fibrillation (SDAF). Among patients with SDAF (<30 seconds, n = 207), 80.2% had a CHA₂DS₂-VASc score ≥2 and 48.8% had scores≥5, compared with 70.3% and 22.4%, respectively, in patients without AF (both*p*< 0.001). There was no significant difference in median CHA₂DS₂-VASc score between patients with SDAF and those with AF ≥30 seconds (median 4 [IQR 3–5] in both groups;*p*= 0.58)

Patients with AF of any duration had significantly higher premorbid CHA₂DS₂-VASc scores than those without AF (median 4 [IQR 3–5] vs. 3 [IQR 2–4]; *p* < 0.001). A score ≥ 2 was present in 91.4% of AF patients versus 70.3% without AF, and scores ≥ 5 were observed in 51.8% vs. 22.4% (both *p* < 0.001) (Fig. 5). No significant difference was observed between long- and short-duration AF (median score 4 [IQR 3–5] in both; score ≥ 5 in 48.8% vs. 51.5%; *p* = 0.58). Among short-duration AF patients (*n* = 207), 166 (80.2%) had scores ≥ 2 and 101 (48.8%) had scores ≥ 5. Low-risk scores (0–1) were found in 18 patients (8.7%), primarily within the small-vessel subgroup. The proportion of CHA₂DS₂-VASc < 2 was higher in small-vessel stroke than in other subtypes (40% vs. 8.1%; *p* = 0.002). In sex-stratified analysis, women with short-duration AF had significantly higher scores than men (median 5 [IQR 4–6] vs. 4 [IQR 3–5]; *p* < 0.001).

### Clinical distinction between known and newly detected AF

Among the 384 patients with AF of any duration, 126 (32.8%) had a documented history of AF prior to stroke onset (premorbid AF), while 258 (67.2%) were newly diagnosed during in-hospital ECG monitoring (AF after stroke). Short-duration AF (< 30 s) was significantly more common in patients with newly detected AF compared to those with premorbid AF (72.1% vs. 18.3%; OR, 11.5; 95% CI, 6.7–19.7; *P* < 0.001). In contrast, AF ≥ 30 s occurred more frequently in patients with premorbid AF (81.7% vs. 27.9%; OR, 10.9; 95% CI, 6.3–18.8; *P* < 0.001).

Stroke severity at admission, assessed using the NIHSS, was significantly lower in patients with newly detected AF than in those with premorbid AF (median NIHSS 4 [IQR, 2–7] vs. 7 [IQR, 4–12]; *P* = 0.002). Among patients with ESUS, all AF episodes were newly detected after stroke, and 35.7% had SDAF. These findings support the notion that AF after stroke may represent a distinct, frequently subclinical and less severe phenotype, underscoring the need for refined post-stroke AF classification and individualized management.

### Premorbid antithrombotic therapy and atrial fibrillation subtype

Premorbid antithrombotic use was prevalent in this cohort, with 94.9% of patients receiving antithrombotic therapy prior to stroke onset. Among patients with coronary or peripheral artery disease, acetylsalicylic acid (ASA) monotherapy was near-universal (> 90%). In contrast, antiplatelet use among patients without known vascular disease was markedly lower (49.5%; OR, 12.3; 95% CI, 7.0–21.6; *P* < 0.001), underscoring the strong influence of documented atherosclerotic disease on preventive strategies.

Among patients with short-duration atrial fibrillation (SDAF), premorbid ASA use was disproportionately high (71.5%) compared to AF-negative patients (53.1%; OR, 2.2; 95% CI, 1.6–3.2; *P* < 0.001), suggesting a partial recognition of underlying vascular or embolic risk. Nevertheless, SDAF patients experienced a median NIHSS of 4 (IQR, 2–8), indicating that ASA may be insufficient in mitigating embolic stroke severity in this group.

AF detection patterns differed markedly between known and newly diagnosed cases. Of 384 patients with AF of any duration, 67.2% were diagnosed post-stroke, predominantly with SDAF (< 30 s; 72.1% vs. 18.3% in known AF; OR, 11.5; 95% CI, 6.7–19.7; *P* < 0.001). Conversely, sustained AF was more common in the premorbid AF group (81.7% vs. 27.9%; OR, 10.9; 95% CI, 6.3–18.8; *P* < 0.001), correlating with higher anticoagulation rates (87.3%) prior to stroke. Among older patients with known AF, NOACs were the predominant anticoagulant choice (91.2%), while VKAs were limited to patients with specific comorbidities.

Strikingly, none of the patients with newly detected AF were anticoagulated at stroke onset (0.0% vs. 87.3%; OR, ∞; 95% CI, 49.9–∞; *P* < 0.001), yet this group exhibited lower stroke severity (median NIHSS 4 [IQR, 2–7]) compared to those with known AF (median NIHSS 7 [IQR, 4–12]; *P* = 0.002). This contrast suggests divergent pathophysiologic mechanisms and risk profiles between incident and chronic AF, with potential implications for post-stroke rhythm monitoring and treatment thresholds.

Among patients with prior cerebrovascular events (*n* = 181), 97.8% were receiving secondary prevention at baseline—primarily ASA (80.7%) or NOACs (17.1%). Despite this, recurrent ischemic events occurred, particularly in those with underlying AF (35.4%), including 39 with SDAF. This subgroup had elevated CHA₂DS₂-VASc scores and moderate stroke severity, underscoring the limitations of current antithrombotic strategies and the need for refined post-stroke risk stratification, especially in patients with occult or subclinical AF.

### Exploratory multivariable regression analysis

To explore factors independently associated with short-duration atrial fibrillation (SDAF), we conducted a multivariable logistic regression including age > 65 years, female sex, hypertension, CHA₂DS₂-VASc score, and stroke subtype (with ESUS as reference category). SDAF was used as the dependent variable (1 = SDAF present, 0 = no AF or only longer-duration AF).

In the final model, age > 65 years (OR 3.7; 95% CI 2.4–5.9; *p* < 0.001), female sex (OR 1.9; 95% CI 1.4–2.7; *p* < 0.001), and hypertension (OR 1.8; 95% CI 1.2–2.7; *p* = 0.004) emerged as independent predictors of SDAF. A higher CHA₂DS₂-VASc score was also significantly associated with the presence of SDAF (per point increase: OR 1.4; 95% CI 1.2–1.6; *p* < 0.001). Importantly, the ESUS subtype remained significantly associated with SDAF compared to other stroke categories (OR 1.5; 95% CI 1.0–2.3; *p* = 0.049). Full model results are presented in Supplementary Table 1. These findings support the descriptive results and suggest that traditional vascular risk factors and embolic stroke subtype are independently associated with the detection of short-duration atrial fibrillation.

### Echocardiographic characteristics by SDAF status

Transthoracic echocardiography was available for all patients. Patients with short-duration atrial fibrillation (SDAF) exhibited a distinct atrial profile compared to those without SDAF. Notably, left atrial volume index (LAVI) was significantly higher in the SDAF group (mean 39.5 ± 10.6 mL/m² vs. 33.4 ± 9.1 mL/m²; *p* = 0.002), and atrial reservoir strain was markedly reduced (–17.1 ± 4.3% vs. − 21.5 ± 5.2%; *p* = 0.001). Measures of diastolic function, including E/e′ ratio and LAVI/a′ ratio, also differed significantly between groups. While left ventricular ejection fraction (LVEF) was numerically lower in the SDAF group, this difference did not reach statistical significance. Similarly, mitral regurgitation ≥ mild was more common among SDAF patients, though the association was not statistically significant (*p* = 0.083). These findings suggest a subclinical atrial cardiomyopathy pattern in patients with SDAF. (*Corresponding data are summarized in* Table 2*)*

**Table 2:** Echocardiographic characteristics by presence of Short-Duration atrial fibrillation this table summarizes echocardiographic differences between patients with and without SDAF. Those with SDAF had significantly larger left atria, higher LAVI, impaired atrial strain, and more advanced diastolic dysfunction, while LVEF and mitral regurgitation rates were similar between groups. Values are presented as mean ± sd or number (percentage)

Echocardiographic parameter	SDAF present (*n* = 154)	SDAF absent (*n* = 375)	*p*-value
Left ventricular ejection fraction, % (mean ± SD)	58.3 ± 7.1	59.8 ± 6.3	0.117
Left atrial enlargement ≥ 42 mm, n (%)	105 (68.2%)	159 (42.5%)	<** 0.001**
Left atrial volume index (LAVI), mL/m² (mean ± SD)	39.5 ± 10.6	33.4 ± 9.1	**0.002**
Septal PA-TDI, ms (mean ± SD)	131.2 ± 12.5	102.3 ± 10.2	< **0.001**
LAVI/a′ ratio (mean ± SD)	4.3 ± 1.1	3.1 ± 0.9	**0.004**
Diastolic dysfunction ≥ grade II, n (%)	64 (41.5%)	96 (25.6%)	**0.009**
E/e′ ratio (mean ± SD)	12.8 ± 4.1	10.4 ± 3.7	**0.006**
Mitral regurgitation ≥ mild, n (%)	41 (26.5%)	67 (17.8%)	0.083
Left atrial strain, % (mean ± SD)	–17.1 ± 4.3	–21.5 ± 5.2	**0.001**

Values are presented as mean ± SD or number (percentage), as appropriate

Bold *p*-values indicate statistically significant differences (p < 0.05)

*LAVI* Left atrial volume index, *LA Strain* Left atrial reservoir strain, *E/e*′ Ratio of early mitral inflow velocity to mitral annular early diastolic velocity

## Discussion

This study identifies a clinically important subgroup of stroke and TIA patients with short-duration atrial fibrillation (SDAF < 30 s) who remain undetected or untreated under current diagnostic thresholds. Although these brief episodes fall below the guideline-defined cut-off for atrial fibrillation [9, 17, 18], they were observed in nearly one-third of our cohort—particularly among patients with embolic stroke of undetermined source and cryptogenic stroke. Most affected individuals had elevated CHA₂DS₂-VASc scores, mild to moderate stroke severity, and a disproportionate representation of older adults and women. These findings suggest that SDAF may represent an overlooked marker of embolic potential, highlighting a diagnostic blind spot in current stroke evaluation.

In this prospective cohort of patients with ischemic stroke or TIA, short-duration AF (< 30 s) was detected in 29% of all cases and 35.7% of ESUS. Over 80% of these patients had CHA₂DS₂-VASc scores ≥ 2, and nearly half had scores ≥ 5, indicating substantial thromboembolic risk despite not meeting the diagnostic AF threshold of ≥ 30 s **(**Figs. 1 and 5). NIHSS scores were significantly higher among patients with elevated CHA₂DS₂-VASc scores, and a modest but significant correlation between stroke severity and thromboembolic risk was observed (ρ = 0.23, *p* = 0.001). Higher CHA₂DS₂-VASc scores are independently associated with increased embolic risk even in patients without atrial fibrillation, with a consistent stepwise increase in risk across large population-based studies [19, 20]. Nonetheless, its moderate discriminatory power limits clinical utility, and current guidelines do not support its use for anticoagulation decisions in non-AF populations. Our findings reinforce this association, demonstrating that elevated CHA₂DS₂-VASc scores may reflect relevant embolic risk even in patients without guideline-defined AF [21].

In light of this, the adequacy of the 30-second threshold for defining clinically relevant AF may require re-evaluation in high-risk patients with brief arrhythmic episodes. While not all short AF episodes require treatment, the 30-second threshold remains a long-established diagnostic convention endorsed by major societies including the ESC, AHA/ASA, and HRS/EHRA [2, 12, 22, 23, 24]. This cutoff has been consistently applied in key stroke trials such as CRYSTAL-AF, EMBRACE, and STROKE-AF [25–27] and is used to guide post-stroke rhythm monitoring and classification in international and national guidelines. In our study, the 30-second threshold was used solely for detection and classification—not to imply a treatment indication. We also acknowledge that in real-world device-based monitoring, episode durations ≥ 6 min are commonly used to guide oral anticoagulation, as recently demonstrated in the ARTESiA trial [28]. Our findings aim to inform early risk stratification and generate hypotheses, not to redefine treatment thresholds. Emerging evidence suggests that even AF episodes < 30 s may predict future clinically manifest AF and should not be dismissed as benign—particularly in high-risk stroke populations [29, 30].

**Fig. 6:**
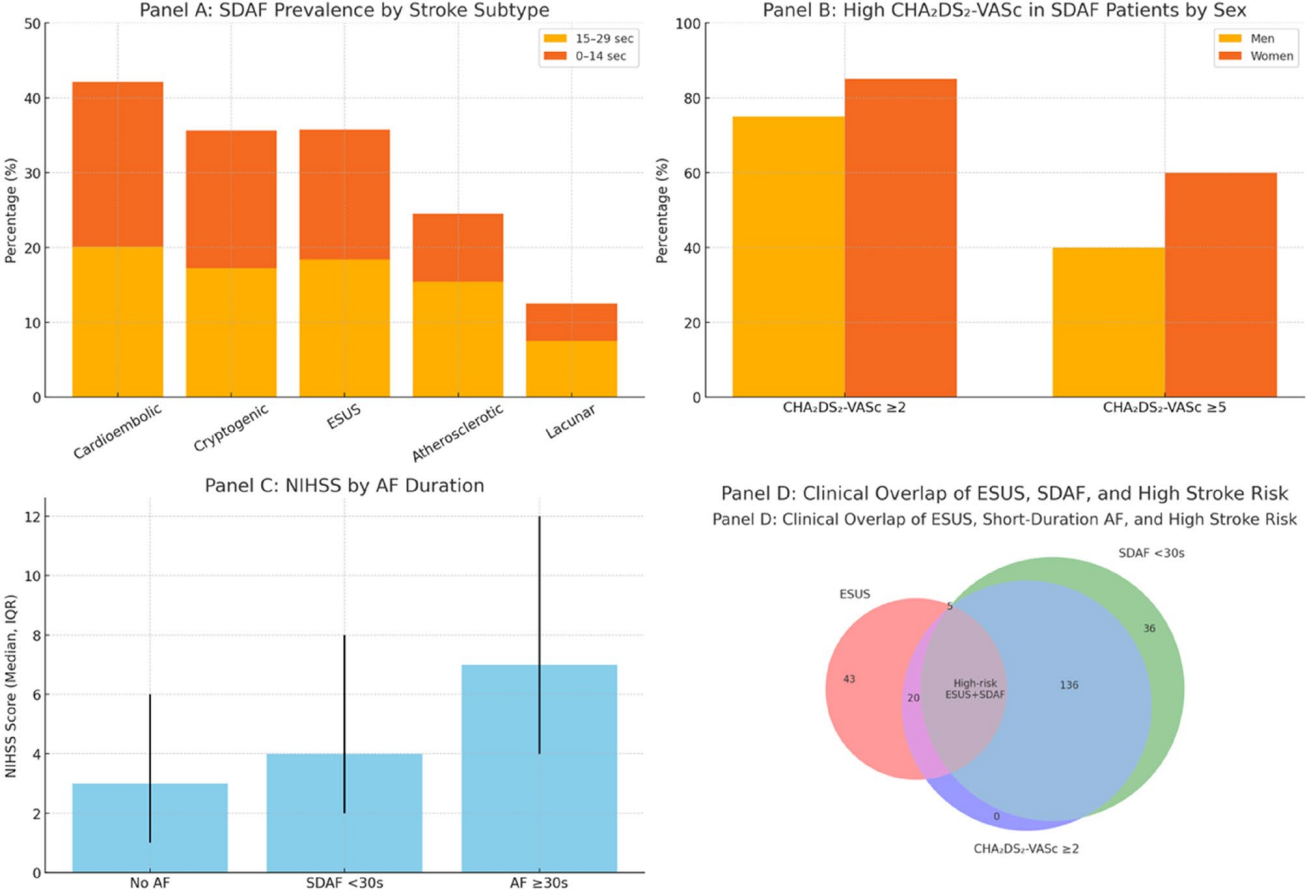
Short-Duration Atrial Fibrillation in Ischemic Stroke: Subtype Distribution, Risk Stratification, and Clinical Overlap.**Panel A** SDAF Prevalence by Stroke Subtype Stacked bar chart showing the prevalence of SDAF episodes of 0–14 seconds and 15–29 seconds across stroke subtypes. SDAF was most common in cardioembolic strokes, followed by cryptogenic and ESUS, and least common in lacunar strokes.**Panel B** High CHA₂DS₂-VASc in SDAF Patients by Sex Bar chart comparing the proportion of SDAF patients with CHA₂DS₂-VASc scores ≥2 and≥5 by sex. A higher percentage of women with SDAF had CHA₂DS₂-VASc≥5, indicating elevated stroke risk despite short AF duration.**Panel C** NIHSS by AF Duration Bar chart depicting the median NIHSS score (with interquartile range) at admission by AF status. Stroke severity was highest in patients with AF ≥30 seconds, followed by SDAF <30s, and lowest in those without AF.**Panel D** Clinical Overlap of ESUS, SDAF, and High Stroke Risk Venn diagram illustrating the intersection of ESUS, SDAF <30s, and CHA₂DS₂-VASc≥2. A notable subset of patients exhibit all three features, highlighting a high-risk ESUS group with occult atrial fibrillation and elevated stroke risk

The ESUS classification seeks to identify embolic strokes without an identifiable cause, yet our findings demonstrate that short-duration AF is common in both ESUS and cryptogenic strokes. While the overall difference across stroke subtypes was statistically significant (*p* < 0.001), the higher SDAF prevalence in ESUS compared to large-artery stroke did not reach statistical significance (*p* = 0.08), suggesting a possible trend. Only 23% of our cohort met the guideline-defined AF threshold (≥ 30 s), while a larger subset had shorter AF episodes. Among ESUS patients with short-duration AF, 80% had CHA₂DS₂-VASc ≥ 2 and nearly 50% had scores ≥ 5, suggesting a considerable embolic risk. Prior work indicates that CHA₂DS₂-VASc scores > 2 are predictive of incident AF, [31, 32] supporting the idea that these brief arrhythmias may reflect a clinically relevant underlying substrate [33]. Short-duration AF disproportionately affected older adults and women—groups known to carry a higher baseline stroke risk. While these findings offer meaningful insights into the prevalence and clinical profile of short-duration AF in ischemic stroke, they should be interpreted in the context of the study’s observational design. Although causality cannot be firmly established, the associations observed are clinically relevant and underscore the need for prospective studies to further explore the potential pathophysiological and therapeutic implications of these brief arrhythmias. In our study, 89.4% of patients with short-duration AF were over 65 years, and over two-thirds were 75 or older. Women were more frequently affected than men (37.0% vs. 22.8%; OR 2.0; 95% CI, 1.4–2.9; *p* < 0.001) and had more severe strokes, with a higher median NIHSS on admission (5 [IQR 3–10] vs. 3 [IQR 2–7]; *p* = 0.002), in line with epidemiologic data showing rising AF prevalence with age and greater stroke vulnerability in women [14, 31]. These findings raise concerns about the recent removal of female sex as a standalone CHA₂DS₂-VASc component [34, 35]. Additionally CHA₂DS₂-VASc scores correlated significantly with stroke severity (ρ = 0.23; *p* = 0.001), indicating a non-negligible risk of recurrence even in patients without guideline-defined AF. Stroke severity also varied by AF status and stroke subtype. Cardioembolic strokes were associated with the highest NIHSS scores and most severe deficits. Patients with AF—regardless of episode duration—had significantly more severe strokes than those without AF (Fig. 6).

Notably, the severity patterns observed in patients with short-duration AF did not clearly mirror those of classic cardioembolic strokes. Instead, the median NIHSS scores in this group fell within a moderate range and more closely resembled the distribution seen in atherosclerotic strokes (Fig. 4). This suggests that stroke severity alone may not reliably distinguish embolic mechanisms in the presence of SDAF and supports the view that ESUS encompasses heterogeneous pathophysiologies—including occult AF and non-stenotic atherosclerosis.

The RE-SPECT ESUS and NAVIGATE ESUS trials failed to demonstrate a benefit of DOAC therapy in unselected ESUS populations [36, 37] but subgroup analyses by AF duration were not performed. Given that 30–33% of cryptogenic and ESUS strokes in our study exhibited short-duration AF, this subgroup may merit separate consideration in future anticoagulation trials. Prolonged ECG monitoring, cardiac imaging, and biomarkers may improve detection of AF-related stroke risk. The SAFAS study showed that multimodal diagnostic approaches enhance post-stroke AF detection [38]. According to current studies, AF predictors in ESUS include older age, high CHA₂DS₂-VASc scores, rhythm irregularity burden (HR 3.12), elevated NT-proBNP, left atrial enlargement, NSAT, prolonged PR interval, and specific imaging patterns (e.g. non-lacunar, bihemispheric, multifocal) [17, 39, 40]. and elevated CHA₂DS₂-VASc and CHA₂DS₂ scores have been linked to delayed post-stroke AF onset [31, 41]. Recent data from the ARTESIA trial demonstrated that anticoagulation with apixaban reduces stroke risk in patients with subclinical AF lasting 6 min to 24 h, albeit with an increased bleeding risk [28]. A subgroup analysis found particular benefit in patients with prior stroke or TIA and subclinical AF, demonstrating potential benefits of individualized anticoagulation decisions based on risk profile rather than fixed duration thresholds [42, 43]. 

Our findings identified a clinically relevant subgroup of stroke patients with short-duration AF and elevated risk profiles. Future studies should explore the prognostic relevance and long-term outcomes of SDAF in high-risk stroke populations. Our results underscore the need for burden-adapted follow-up strategies that account for arrhythmia duration, patient risk profile, and stroke subtype.

### Limitations and strengths

This single-center design may limit generalizability. Data on prior anticoagulant or antiplatelet therapy were not available, and long-term follow-up was not performed, precluding assessment of stroke recurrence or AF progression. Strengths include the prospective design, standardized data collection, and structured neurological assessments during hospitalization. Daily clinical evaluation minimized missing data, and detailed ECG analysis enhanced detection and classification of atrial fibrillation. As with all descriptive observational studies, causality cannot be inferred; however, the consistent patterns observed in high-risk subgroups underscore the need for prospective validation, to see whether or not short-duration AF is a predictor for developing longer episodes of AF or recurrent strokes. Furthermore, the use of a 30-second threshold for AF detection, while standardized in trials and guidelines, may not align with real-world treatment practices, which often apply longer duration cutoffs (e.g., ≥ 6 min) when considering anticoagulation initiation.

## Conclusions

Short-duration atrial fibrillation occurred across all ischemic stroke subtypes, with the highest prevalence in cardioembolic, cryptogenic, and ESUS cases. It was more frequent in older adults and women and associated with elevated CHA₂DS₂-VASc scores, indicating substantial thromboembolic risk despite falling below current AF diagnostic thresholds. These findings suggest that SDAF may signal a clinically relevant embolic source and define a high-risk, undertreated subgroup.

This diagnostic blind spot underscores the need to reconsider fixed duration thresholds in post-stroke AF detection. Risk-based approaches incorporating age, sex, and CHA₂DS₂-VASc may better guide monitoring and secondary prevention. Prospective trials are needed to determine whether targeted treatment of SDAF can improve outcomes in this overlooked population.

## Supplementary Information

Supplementary Material 1.

## Data Availability

The datasets generated and/or analysed during the current study are available from the corresponding author on reasonable request.
