# Supplementary material for: Short-duration atrial fibrillation in ischemic stroke: high risk despite subclinical burden-a prospective cohort study
Source: BMC Cardiovasc Disord. 2025 Aug 20;25:616. doi: 10.1186/s12872-025-05080-1 (PMC12369059; doi:10.1186/s12872-025-05080-1)
Supplement: Supplementary file 1 — Supplementary Material 1. [file 12872_2025_5080_MOESM1_ESM.docx]

**Supplementary Table 1. Multivariable logistic regression predicting short-duration atrial fibrillation (SDAF)**
*n = 714 patients; 207 with SDAF (29.0%)*

| **Variable** | **OR** | **95% CI** | **p-value** |
| --- | --- | --- | --- |
| Age > 65 years | 3.7 | 2.4 – 5.9 | <0.001 |
| Female sex | 1.9 | 1.4 – 2.7 | <0.001 |
| Hypertension | 1.8 | 1.2 – 2.7 | 0.004 |
| CHA₂DS₂-VASc (per point) | 1.4 | 1.2 – 1.6 | <0.001 |
| ESUS (vs. cardioembolic) | 1.5 | 1.0 – 2.3 | 0.049 |
